# Efficacy and safety of electroacupuncture for carpal tunnel syndrome (CTS): A systematic review and meta-analysis of randomized controlled trials

**DOI:** 10.3389/fsurg.2022.952361

**Published:** 2022-09-23

**Authors:** Ting Li, Jingxin Yan, Jiang Hu, Xilin Liu, Fei Wang

**Affiliations:** ^1^ Department of Orthopedics, Sichuan Provincial People's Hospital, Chengdu, China; ^2^ Department of Postgraduate, Chengdu Medical College, Chengdu, China; ^3^ Department of Interventional Therapy, Affiliated Hospital of Qinghai University, Xining, China; ^4^ Department of Postgraduate, Qinghai University, Xining, China

**Keywords:** meta-analysis, carpal tunnel syndrome, electroacupuncture, effect, systematic review

## Abstract

**Aim:**

We carried out a systematic review and meta-analysis to evaluate the safety and efficacy of electroacupuncture for patients with carpal tunnel syndrome.

**Methods:**

We searched PubMed, Embase, Cochrane Library, Scopus, Web of Science, Chinese National Knowledge Infrastructure (CNKI), Chongqing VIP Database (VIP), and Wan Fang Database up to May 2022 for relevant studies. Relevant studies were identified by using specific eligibility criteria and data were extracted.

**Results:**

A total of 26 randomized controlled trials (RCTs) with 1,698 patients were included. Compared with routine treatment, electroacupuncture treatment had lower visual analog scale (VAS) score [mean difference* *=* *−0.79, 95% confidence interval (CI): −1.11 to −0.47, *P *< 0.00001], and the symptom severity scale and function status scale in electroacupuncture group were significantly lower than the control group (*P *= 0.0001 and *P *= 0.006). Moreover, the electrophysiological parameters in the electroacupuncture group were better than the control group. The electroacupuncture group had higher total effective rate than the control group (odds ratio* *= 4.94, 95% CI: 3.44–7.08, *P *< 0.00001).

**Conclusion:**

Our meta-analysis indicated that electroacupuncture had lower VAS score, higher total effective rate, a lower the scores of symptoms and function and electroacupuncture had better electrophysiological parameters. However, these findings needed to be verified further by multicenter, double-blind, and large-sample RCTs.

## 1. Introduction

Carpal tunnel syndrome (CTS) is caused by compression of the median nerve. CTS adversely affects daily activities and affects general health and quality of life. The prevalence of CTS has been reported to be 36 per 10,000 people, and in the United States, approximately 25 women and 13 men per 10,000 adults receive carpal tunnel release surgery per year (1–3). CTS is diagnosed by clinical symptoms (such as discomfort, paraesthesia, and numbness) and electrophysiological diagnostic tests (4, 5). Occupational risk factors have long been recognized, including repetitive, forceful hand work, wrist extension and vibration, and cold conditions (6).

Currently, standard surgical and nonsurgical strategies for treating CTS have been reported, but the safety and efficacy are still unclear (7). Mild-to-moderate degree CTS patients often opt for nonsurgical treatments. Nonsurgical treatments include laser therapy, pharmacotherapy, therapeutic ultrasound, musculoskeletal manipulation, splinting, and acupuncture (4, 8, 9). Electroacupuncture treatment can relieve pain by activating a variety of bioactive chemicals through peripheral, spinal, and supraspinal mechanisms, and a review reported that electroacupuncture might be more effective in relieving pain than standard manual acupuncture (10, 11).

However, some studies demonstrated that electroacupuncture treatment is not superior to acupuncture in improving symptomatic. On the contrary, control treatment can exert positive therapeutic effects for CTS patients, as evidenced by improved symptomatology, electrophysiological function, and physical provocation sign (12). Hence, electroacupuncture treatment is still questioned. We aim to conduct a systematic review and meta-analysis of the current literature to assess the relative benefits and risks of electroacupuncture treatment compared with control treatment.

## 2. Materials and methods

### 2.1 Study design

This meta-analysis is in accordance with the PRISMA statement (13).

### 2.2 Literature retrieval strategy

Electronic databases such as PubMed, Embase, Cochrane Library, Scopus, UpToDate, Web of Science, Chinese National Knowledge Infrastructure (CNKI), Chongqing VIP Database (VIP), and Wan Fang Database were searched up to May 2022. All randomized controlled trials (RCTs) that used electroacupuncture treatment for carpal tunnel syndrome were collected. The retrieval method adopted the combination of subject words and free words, and English retrieval words and Chinese versions include ((((((((Carpal Tunnel Syndromes[Title/Abstract]) OR (Syndrome, Carpal Tunnel[Title/Abstract])) OR (Amyotrophy, Thenar, Carpal Origin[Title/Abstract])) OR (Median Neuropathy, Carpal Tunnel[Title/Abstract])) OR (Compression Neuropathy, Carpal Tunnel[Title/Abstract])) OR (Entrapment Neuropathy, Carpal Tunnel[Title/Abstract])) OR (CTS[Title/Abstract])) OR (“Carpal Tunnel Syndrome”[Mesh])) AND ((Acupuncture[Title/Abstract]) OR (“Electroacupuncture”[Mesh])); in addition, the references of the included literature were reviewed to supplement the relevant studies. Ethical approval for meta-analysis was not required because meta-analysis did not involve any subject directly.

### 2.3 Inclusion and exclusion criteria

#### 2.3.1 Inclusion criteria

Studies were eligible for inclusion if they met the following criteria of PICOS: (1) P: patients with primary CTS who did not undergo surgery were included. (2) I: patients with CTS were treated by electroacupuncture. (3) C: other treatments for patients with CTS. (4) O: the clinical outcomes included visual analog scale (VAS) pain scores, symptom severity scale and function status scale, electrophysiological parameters [including median nerve distal motor latency (DML), compound muscle action potentials amplitude (CMAP), sensory conduction velocity (SCV), and sensory nerve action potential (SNAP)], and total effective rate. (5) S: all RCTs were included.

#### 2.3.2 Exclusion criteria

Studies were ineligible if they met the following criteria: (1) studies could not extract data studies so that the study could not be analyzed; (2) duplicate reports; (3) patients with CTS undergone surgical treatment; (4) relevant outcome indexes were not reported; (5) animal studies, biomechanical studies, duplicate publications of one trial, case report, letter, revision, technology note, commentaries, reviews, withdraw trails, and meta-analysis.

### 2.4 Data extraction

Two researchers independently read the full text of potential studies that met the inclusion and exclusion criteria. The data were extracted as follows: basic information on the sample included in the study (year of publication, total number of participants, authors, age, gender, intervention, etc.), study design type (prospective and retrospective study), study duration, and study observation indicators. Moreover, we extracted the following data from each selected study: (1) VAS pain scores: VAS scoring system was global observational rating scales and was used to evaluate the pain level of patients. Patients mark the location on the 10-cm line corresponding to the amount of pain. 0 was no pain and 10 was severest pain. (2) Symptom severity scale and function status scale: the Levine Questionnaire (also known as the Boston questionnaire) is regarded as a valuable assessment of severity of symptoms and functional status in carpal tunnel syndrome. The symptoms severity scale has 11 questions. The functional status scale has eight questions. A summary score of 1 (mildest) to 5 (most severe) is obtained, with a higher score indicating greater symptom severity. (3) Electrophysiological parameters. (4) Total effective rate. When information was missing, we attempted to contact the primary author *via* email to seek clarification or exclude the study.

### 2.5 Risk of bias assessment

The risk of bias in the included studies was evaluated using the Cochrane tool to assess risk of bias for the RCTs (14). Bias assessments were carried out by two independent researchers. Any unresolved disagreements between reviewers were resolved through discussion or through evaluation by a third reviewer.

### 2.6 Statistical analysis

The Revman 5.4 software package was used for this meta-analysis. The dichotomous outcomes were reported by odds ratio (OR) with 95% confidence interval (CI), and we reported continuous outcomes for mean difference (MD) or standardized mean difference (SMD) with 95% CI. Heterogeneity test was performed on the included study results using the chi-square test. If *I*^2^ ≤ 50%, it indicated that there was no homogeneity among the research results, and a fixed effect model was used. If *P* < 0.05 and *I*^2^ > 50%, then heterogeneity existed among studies, and a random effect model was used. We also performed a sensitivity analysis to identify the resource of the heterogeneity. Publication bias was assessed by the funnel plot.

## 3 Results

### 3.1 Search result

**Figure 1:**
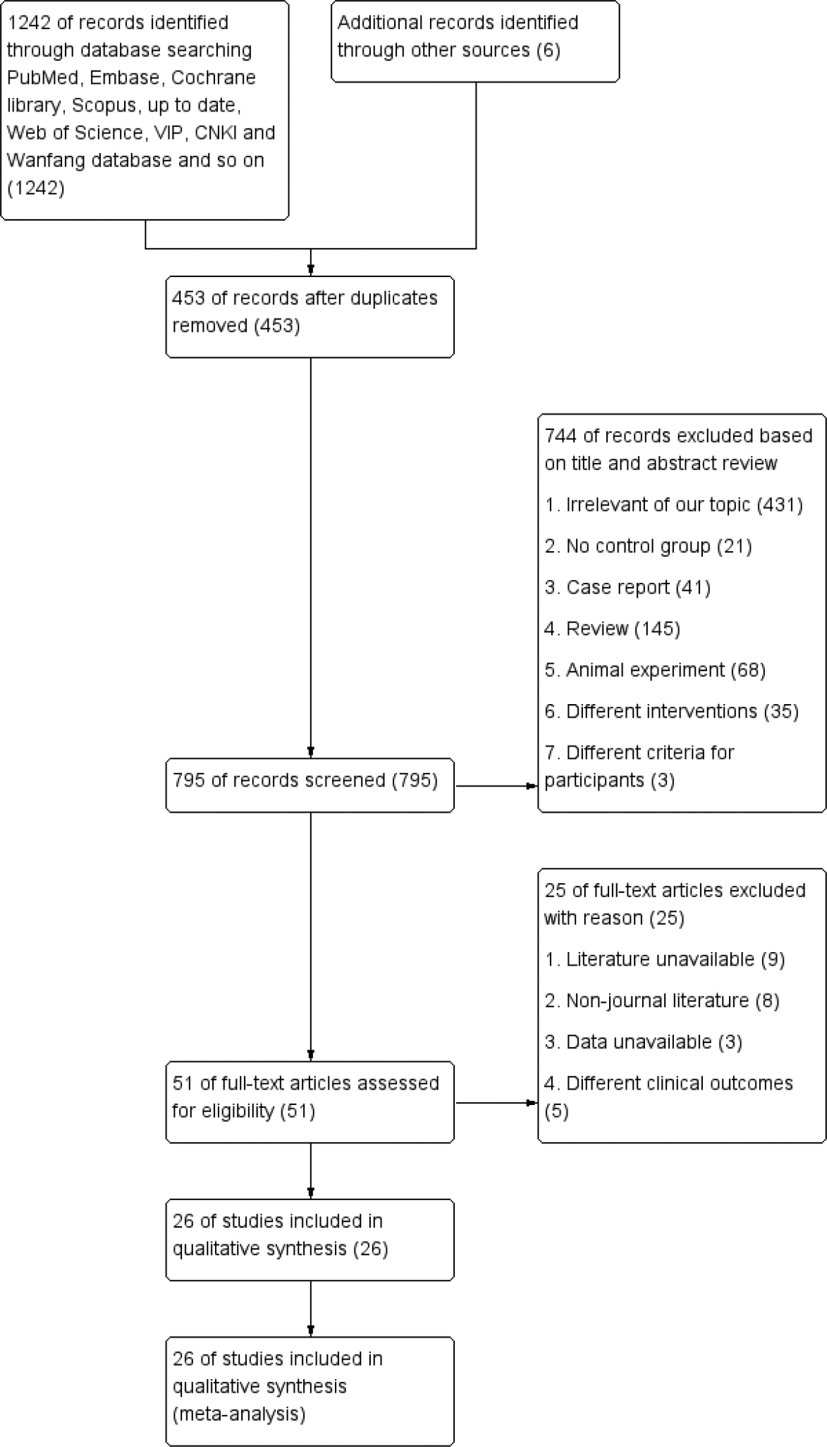
Flowchart of the study.

The initial search yielded 1,248 records where we excluded 453 records due to duplication. After examination of the titles, abstracts, and full-text articles, 26 potentially eligible studies (12, 15–39) were assessed for inclusion criteria. After application of the inclusion criteria, 7 trials published in English and 19 trails published in Chinese were included in this meta-analysis. Figure 1 displays the selection algorithm and the number of included and excluded studies. All titles, abstracts, and text were dually and independently reviewed by the authors based on the inclusion and exclusion criteria to minimize bias.

### 3.2 Study characteristic

A total of 26 RCTs (12, 15–39) included 1,695 patients in this meta-analysis. Among those included studies, 13 studies used acupuncture treatment as a control group, 9 studies used drug treatment as a control group, 2 studies used Neuro tendon gliding training as a control group, and 2 studies used wrist splints as a control group. The main basic characteristics of the included studies are shown in Table 1.

**Table 1:** Basic characteristics of the included literature.

Name	Year	Age (I/C)	Region	Number of persons (I/C)	Intervention group	Controlled group	Inclusion criteria	Follow-up time (I/C)
Ding	2013	57/55.11	China	19/19	Electroacupuncture (2 Hz, 20 min, 5/week, 2 weeks, Yangchi acupoint)	Acupuncture (5/week, 20 min, 2 weeks, Yangchi acupoint)	NA	3 months/ 3 months
Li	2011	42.25/41.03	China	40/40	Electroacupuncture (2–20 Hz, 5–15 V, 20 min, 5/week, 4 weeks, Daling and Neiguan acupoints)	Neurotrophic drugs (4 weeks)	Mild to moderate	1 month/ 1 month
Du	2014	43.5/41.4	China	23/23	Electroacupuncture (30 min, 7/week, 2 weeks, Neiguan, Waiguan, Nei laogong, and Wai laogong acupoints)	Acupuncture by well point bloodletting (2 weeks, Neiguan, Waiguan, Nei laogong, and Wai laogong acupoints)	NA	NA
Yang	2011	51.6/50.7	China	40/40	Electroacupuncture (2–20 Hz, 5–15 V, 20 min, 5/week, 4 weeks, Daling and Neiguan acupoints)	Chinese medicine wash (20 min, 5/week, 4 weeks)	Mild to moderate	1 month/ 1 month
Zeng	2014	59.3/56.7	China	29/29	Electroacupuncture (30 min, 10 days, Neiguan acupoint)	Acupuncture (NA)	NA	NA
Xie	2018	41.26/41.78	China	43/43	Electroacupuncture (2–20 Hz, 5–15 V, 20 min, 5/week, 4 weeks, Deling and Neiguan acupoints)	Neurotrophic drugs (4 weeks)	Mild to moderate	1 month/ 1 month
Xiang	2014	45.78/46.02	China	30/30	Electroacupuncture (30 min, 6/week, 4 weeks, Shixuan, Daling, Laogong and Neiguan acupoints)	Neurotrophic drugs (4 weeks)	Mild to moderate	1 month/ 1 month
Hu	2018	42.6/42.6	China	50/50	Electroacupuncture (1–100 Hz, 30 min, 6/week, 5 weeks, Daling, Yangxi, Neiguan, and Hegu acupoints)	Acupuncture (30 min, 6/week, 5 weeks, Daling, Yangxi, Neiguan, and Hegu acupoints)	NA	5 weeks/ 5 weeks
Zhao	2012	43.68/43.52	China	25/25	Electroacupuncture (1–100 Hz, 30 min, 6/week, 5 weeks, Daling, Neiguan, Yangxi, Hegu, and Laogong acupoints)	Acupuncture (30 min, 6/week, 5 weeks, Daling, Neiguan, Yangxi, Hegu, and Laogong acupoints)	NA	5 weeks/ 5 weeks
Jin	2011	46/44	China	25/25	Electroacupuncture (30 min, 1/day, 10 days, Daling, Neiguan, Yangxi, Hegu, and Yuji acupoints)	Neurotrophic drugs (20 days)	Mild	3 weeks/ 3 weeks
Ye	2014	46/46	China	32/31	Electroacupuncture (2 Hz, 30 min, 1/day, 10 days, Daling, Hegu, Waiguan, and Quchi acupoints)	Acupuncture (1/day, 10 days, Daling, Hegu, Waiguan, and Quchi acupoints)	NA	NA
Wei	2017	46.3/50.2	China	30/30	Electroacupuncture (30 min, 1/day, 10 days, Quchi, Waiguan, Neiguan, Hegu, Daling, and Yangxi acupoints)	Neurotrophic drugs (10 days)	Mild to moderate	NA
Wu	2014	60.6/58.6	China	15/15	Electroacupuncture (30 min, 1/day, 10 days, Daling, Neiguan, Yuji, and Laogong acupoints)	Wrist splints	NA	NA
Song	2013	42/43.5	China	40/34	Electroacupuncture (30 min, 1/day, 20 days, Neiguan, Daling, Yuji, and Laogong acupoints)	Neurotrophic drugs (20 days)	NA	3 weeks/ 3 weeks
Chen	2021	33.12/31.89	China	42/42	Electroacupuncture (30 min, 5/day, 1 month, Daling, Hegu, Yangxi, Yuji, Laogong, Waiguan, and Neiguan acupoints)	Neurotrophic drugs + physiotherapy (1 month)	Mild to moderate	1 month/ 1 month
Li	2021	42.66/42.25	China	50/50	Electroacupuncture (2–20 Hz, 20 min, 5/week, 4 weeks, Daling and Neiguan acupoints)	Neuro tendon gliding training (7/week)	Mild to moderate	3 months/ 3 months
Shou	2017	43.3/45.4	China	20/20	Electroacupuncture (1 Hz, 20 min, 5/week, 4 weeks, Quchi, Shou sanli, Neiguan, Yangchi, Hegu, and Laogong acupoints)	Neuro tendon gliding training (5/week)	Mild to moderate	4 weeks/ 4 weeks
Chen	2021	65/62.8	China	38/38	Electroacupuncture (45 min, 2/week, 6 weeks, Daling and Neiguan acupoints)	Neurotrophic drugs	Mild to moderate	6 weeks/ 6 weeks
Xiong	2020	46.3/49.2	China	24/24	Electroacupuncture (30 min, 6/week, 6 weeks, Quchi, Neiguan, Daling, Hegu, and Baxie acupoints)	Acupuncture (30 min, 6/week, 6 weeks, Quchi, Neiguan, Daling, Hegu, and Baxie acupoints) + physiotherapy	Mild to moderate	6 weeks/ 6 weeks
Maeda	2017	48.5/50.6	USA	28/23	Electroacupuncture (2 Hz, 20 min, 8 weeks, Daling acupoint)	Acupuncture (8 weeks, Daling acupoint)	Mild to moderate	8 weeks/ 8 weeks
Maeda	2013	49.1/49.1	USA	22/19	Electroacupuncture (2 Hz, 20 min, Daling acupoint)	Acupuncture (Daling acupoint)	NA	NA
Kumnerddee	2010	50.37/51.73	Thailand	30/30	Electroacupuncture (1 Hz, 30 min, 5 weeks, Hegu, Quchi, Daling, Laogong, and Baxie acupoints)	Wrist splints (5 weeks)	Mild to moderate	5 weeks/ 5 weeks
Ho	2014	50.1/49.5	Taiwan	11/15	Electroacupuncture (2 Hz, 15 min, 4/week, 6 weeks, Daling and Neiguan acupoints)	Acupuncture (4/week, 6 weeks, Daling and Neiguan acupoints)	Mild to moderate	6 weeks/ 6 weeks
Salehi	2019	50.75/47.4	Iran	20/20	Electroacupuncture (1 Hz, 40 min, 2/week, 6 weeks, Daling and Neiguan acupoints)	Wrist splints (5 weeks)	Mild to moderate	6 weeks/ 6 weeks
Dimitrova	2019	NA	USA	20/60	Electroacupuncture (2–100 Hz, 20 min, 2 weeks, NA)	Acupuncture (2 weeks, NA)	Mild to moderate	2 weeks/ 2 weeks
Chung	2015	51/51	Hong Kong	90/91	Electroacupuncture (2–100 Hz, 20 min, 2 weeks, NA)	Acupuncture (2 weeks, NA)	Mild to moderate	2 weeks/ 2 weeks

NA, not available.

### 3.3 The bias risk assessment results of the included studies

**Figure 2:**
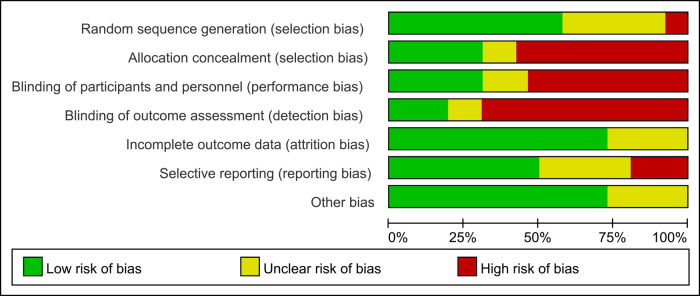
Results of quality assessment using the Cochrane risk tool.

The risk of bias of RCTs was evaluated by the Cochrane tool. The quality assessment of included studies is shown in Figure 2.

### 3.4 Meta-analysis results

#### 3.4.1 Clinical outcomes analysis

##### 3.4.1.1 Visual analog scale

**Figure 3:**
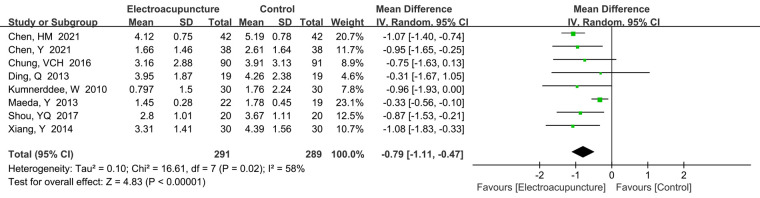
A forest plot showing the VAS score.

A total of eight studies (15, 20, 21, 25, 28, 30, 37, 38) reported the VAS pain score. There was significant heterogeneity (*P *= 0.02, *I*^2^* *=* *58%), so random effects model was performed. We could find that electroacupuncture treatment had lower VAS pain score than the control group (MD* *=* *−0.79, 95% CI: −1.11 to −0.47, *P *< 0.0001; Figure 3). We performed sensitivity analysis to explore the potential source of heterogeneity. By eliminating the included literature one by one, we found that Maeda et al. also had high heterogeneity (*I*^2^* *=* *58% to *I*^2^* *=* *0%).

##### 3.4.1.2 Symptom severity scale

**Figure 4:**
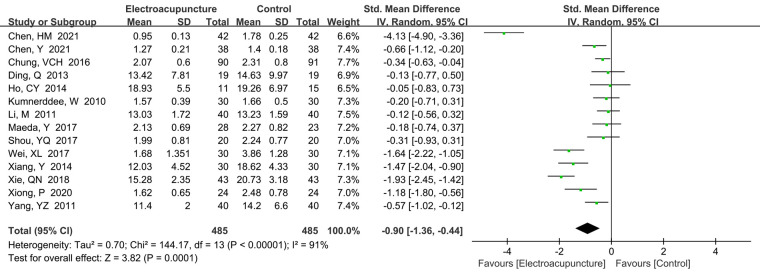
A forest plot showing the function scores.

A total of 14 studies (12, 15, 17, 18, 20, 25, 28–31, 33, 36–38) reported the symptom severity scale. There was significant heterogeneity of studies, so random effects model was performed (*P *< 0.00001, *I*^2^* *=* *91%). We found that electroacupuncture treatment had lower scores than the control group (SMD* *=* *−0.90, 95% CI: −1.36 to −0.44, *P *= 0.0001; Figure 4). We also performed the sensitivity analysis to explore the potential source of heterogeneity, but we found no source of heterogeneity.

##### 3.4.1.3 Function status scale

**Figure 5:**
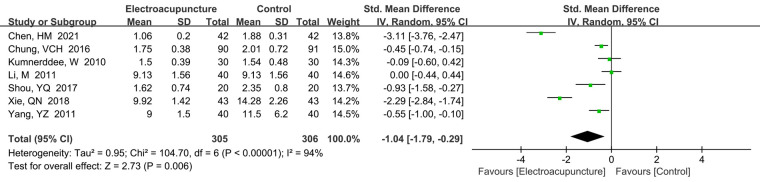
A forest plot showing the scores of symptoms.

A total of seven studies (15, 17, 18, 28, 30, 33, 37) reported the function status scale. There was a high heterogeneity (*P *< 0.00001, *I*^2^* *= 94%). The random effects model was used. The meta-analysis results demonstrated that electroacupuncture treatment had lower function scores than the control group (SMD* *= −1.04, 95%CI: −1.79 to −0.29, *P *= 0.006; Figure 5). We also performed the sensitivity analysis to explore the potential source of heterogeneity, but we also found no the source of heterogeneity.

#### 3.4.2 Electrophysiological parameters analysis

##### 3.4.2.1 Distal motor latency

**Figure 6:**
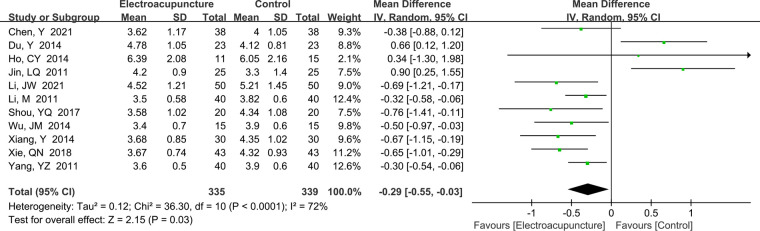
A forest plot showing the DML.

A total of 11 studies (12, 16–18, 23–25, 30, 33, 38, 39) reported the DML. There was significant heterogeneity (*P *< 0.0001, *I*^2^* *= 72%). The random effects model was used. Electroacupuncture treatment in DML was shorter than the control group (MD* *= −0.29 ms, 95% CI: −0.55 to −0.03, *P *= 0.03; Figure 6). Heterogeneity was higher, and the sensitivity analysis did not find the potential source of heterogeneity.

##### 3.4.2.2 CMAP amplitude

**Figure 7:**
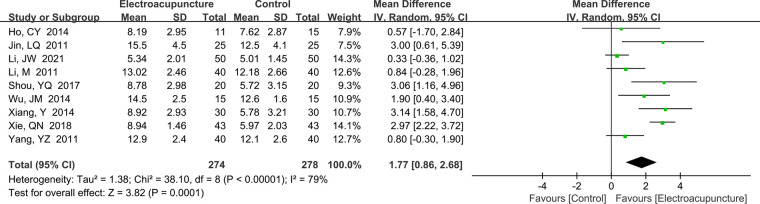
A forest plot showing the CMAP.

A total of nine studies (12, 16–18, 24, 25, 30, 33, 39) reported the CMAP amplitude. There was a high heterogeneity (*P *< 0.00001, *I*^2^* *= 79%). The random effects model was used. The meta-analysis result found that the CMAP amplitude was higher in electroacupuncture treatment than the control group (MD* *= 1.77 mV, 95% CI: 0.86–2.68, *P *= 0.0001; Figure 7). However, we also did not find the heterogeneity by sensitivity analysis.

##### 3.4.2.3 Thumb-carpal SCV and middle finger-carpal SCV

**Figure 8:**
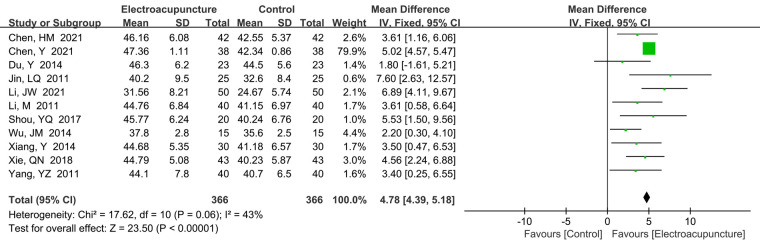
A forest plot showing the thumb-carpal SCV.

A total of 11 studies (16–18, 23–25, 30, 33, 37–39) reported the thumb-carpal SCV. There was no significant heterogeneity (*P *= 0.06, *I*^2^* *= 43%). The fixed effects model was used. Our study demonstrated that electroacupuncture treatment had faster thumb-carpal SCV than the control group (MD* *= 4.78 m/s, 95% CI: 4.39–5.18, *P *< 0.00001; Figure 8).

**Figure 9:**
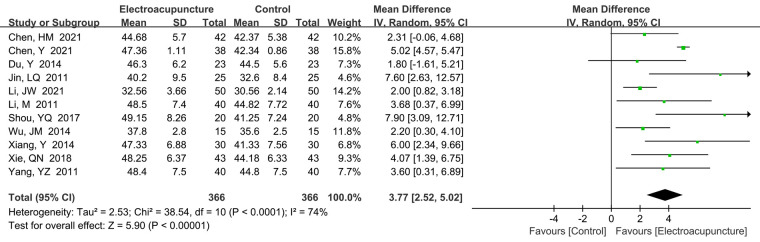
A forest plot showing the middle finger-carpal SCV.

A total of 11 studies (16–18, 23–25, 30, 33, 37–39) reported the middle finger-carpal SCV. There was a high heterogeneity (*P *< 0.0001, *I*^2^* *= 74%). The random effects model was used. We also found that electroacupuncture treatment had faster middle finger-carpal SCV than the control group (MD* *= 3.77 m/s, 95% CI: 2.52–5.02, *P *< 0.00001; Figure 9). We also performed the sensitivity analysis to explore the potential source of heterogeneity. By eliminating the included literature study one by one, we found that Chen et al. also had high heterogeneity (*I*^2^* *= 74% to *I*^2^* *= 39%).

##### 3.4.2.4 Thumb-carpal SNAP amplitude and middle finger-carpal SNAP amplitude

**Figure 10:**
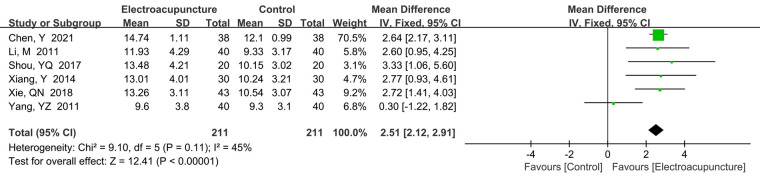
A forest plot showing the thumb-carpal SNAP.

A total of 6 studies (17, 18, 25, 30, 33, 38) reported the thumb-carpal SNAP amplitude. There was no significant heterogeneity (*P *= 0.11, *I*^2^* *= 45%). The fixed effects model was used. In terms of thumb-carpal SNAP, amplitudes significantly higher were obtained in the electroacupuncture treatment (MD* *= 2.51 μV, 95% CI: 2.12–2.91, *P *< 0.00001; Figure 10).

**Figure 11:**
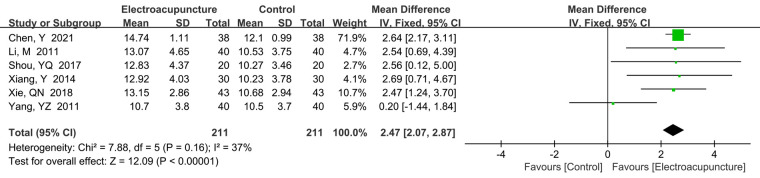
A forest plot showing the middle finger-carpal SNAP.

A total of 6 studies (17, 18, 25, 30, 33, 38) reported the middle finger-carpal SNAP amplitude. There was no significant heterogeneity (*P *= 0.16, *I*^2^* *= 37%). The fixed effects model was used. Our results found that middle finger-carpal SNAP amplitude was higher in the electroacupuncture treatment (MD* *= 2.47 μV, 95% CI: 2.07–2.87, *P *< 0.00001; Figure 11).

#### 3.4.3 Effective rate

##### 3.4.3.1 Total effective rate

**Figure 12:**
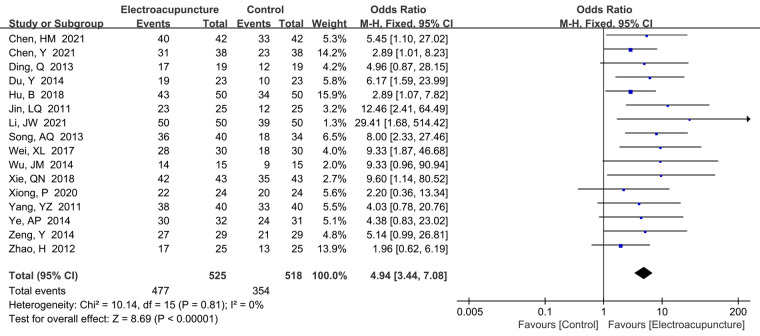
A forest plot showing the total effective rate.

A total of 16 studies (16, 18–20, 22–24, 26, 27, 31–33, 36–39) reported the total effective rate. There was no significant heterogeneity (*P *= 0.81, *I*^2^* *= 0%), so the fixed effects model was used. We found that electroacupuncture treatment had a higher effective rate than the control group (OR* *= 4.94, 95% CI: 3.44–7.08, *P *< 0.00001; Figure 12). Other clinical outcomes could be seen in Table 2.

**Table 2:** Other clinical outcomes of the meta-analysis.

Stratification	No. of studies	No. of patients	Pooled MD/OR	95% CI of pooled MD/OR	*P* value	Heterogeneity *I*^2^ (%)
Crip	2	66	2.81 kg	−0.09 to 5.72	0.06	0
Pinch	2	66	−0.17 kg	−0.89 to 0.55	0.64	0
Recurrence	2	127	0.16	0.07 to 0.36	<0.0001	0
Swelling < 10 mm^2^	2	186	0.25	0.14 to 0.47	<0.0001	0
Complications	3	317	2.32	0.34 to 15.69	0.39	54

MD, mean difference; OR, odds ratio; CI, confidence interval.

### 3.5 Publication bias

**Figure 13:**
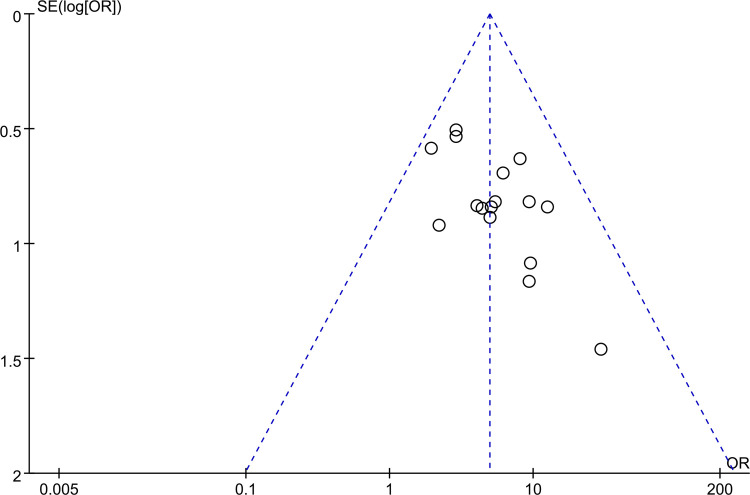
A funnel plot showing publication bias.

The funnel plot was used to evaluate the publication bias of studies. For studies with the total effective rate, the funnel plot was not symmetric (Figure 13). It indicated the possibility of publication bias.

## 4. Discussion

Compressive median neuropathy, also known as CTS, is the most common entrapment neuropathy at the wrist, and tingling, numbness, and pain in the median nerve distribution in the hand are typical symptoms (40). Mild-to-moderate degree CTS usually is treated by nonsurgical therapy such as wrist splinting, corticosteroid injection, acupuncture, and so on, but the evidence on the rest or modification of activity and tendon and nerve gliding regimens is unclear (41). Study reported that low-intensity electroacupuncture was anti-inflammatory and relieved pain by driving the vagal-adrenal anti-inflammatory axis and by inducing vagal activation of aromatic L-amino acid decarboxylase (42, 43). Recently, some studies (34, 35) have reported that electroacupuncture provided better clinical outcomes than other treatments. However, no guideline or consensus was established regarding which type of treatment was preferable to improve clinical outcomes.

The Boston Carpal Tunnel Questionnaire (BCTQ: Symptom Severity Scale and Function Status Scale) and VAS are comparable and these validated clinical outcomes for CTS patients (44, 45). Our meta-analysis demonstrated that electroacupuncture treatment had lower VAS pain score and symptom severity scale and function status scale than the control group. The total score of symptom severity scale and function status scale was calculated, with a higher score indicating greater symptom severity, so we believed that electroacupuncture treatment could improve the patient's symptoms and function. Unfortunately, because of the small size and high heterogeneity for the symptom severity scale and function status scale, the results of studies included in our meta-analysis were of low quality. A clinical trial reported that electroacupuncture reduced pain severity by 1.9 points than the control group (*P* < 0.001) (46). Maeda et al. reported that VAS scores for paresthesia showed significant reductions for electroacupuncture but not for the sham group (electroacupuncture: −1.3 ± 1.6, *P* < 0.001; sham: 0.2 ± 3.5, *P* = 0.82) (21). Moreover, a randomized clinical trial also demonstrated that there was lower VAS score in the electroacupuncture group than the control group, and this clinical trial also found that in the electroacupuncture group, the mean of the symptom severity scale and function status scale decreased significantly (*P* < 0.05); on the contrary, in the control group, symptom severity scale and function status scale did not show significant improvements (*P* = 0.154 and 0.273, respectively) (15). Similarly, Chung et al. also reported that the electroacupuncture group showed greater improvements than the control group at 17 weeks in symptom severity scale and function status scale (*P* = 0.02 and 0.01, respectively) (28). Our meta-analysis also demonstrated that electroacupuncture was superior to the control group, and we performed sensitivity analysis to explore the potential source of heterogeneity. We found that the study by Maeda et al. had high heterogeneity for VAS score (*I*^2^* *= 58% to *I*^2^* *= 0%), but we found no source of heterogeneity for symptom severity scale and function status scale. We believed that this heterogeneity might come from race, different control methods, treatment time, and follow-up time.

Electroacupuncture treatment targets local oxidative injury by enhancing endogenous Nrf2-mediated antioxidative mechanism to relieve pain and inflammation (47). Some studies have found that acupuncture and electroacupuncture can promote nerve regeneration and improve nerve function (48, 49). McCaig et al. demonstrated that electroacupuncture might promote nerve regeneration by an increase in calcium concentration, leading to the activation of nerve outgrowth and repair (50). Our meta-analysis demonstrated that electroacupuncture had shorter DML (median nerve distal motor latency), higher CMAP (compound muscle action potentials amplitude), faster thumb-carpal SCV (sensory conduction velocity), faster middle finger-carpal SCV, higher thumb-carpal SNAP (sensory nerve action potential), and faster middle finger-carpal SNAP. Similarly, Ho et al. also found that electroacupuncture treatment could improve symptomatology, grip strength, and electrophysiological function, and this study also reported that the median motor distal amplitude was significantly increased (*P* = 0.02) and median sensory latency was significantly shortened (*P* = 0.04) (12). In addition, some studies reported that electroacupuncture could increase hand grip strength (51, 52). However, our results demonstrated that there was no significant difference between the electroacupuncture group and the control group for grip and pinch clinical outcomes. According to our meta-analysis, we infer that electroacupuncture could improve electrophysiological parameters but could not increase grip and pinch clinical outcomes.

The results of this meta-analysis showed that electroacupuncture is significantly more effective than control treatment (OR* *= 4.94, 95% CI: 3.44–7.08, *P *< 0.00001). Moreover, we also found that electroacupuncture had lower recurrence rate than control, and the radio of proximal swelling of median nerve of carpal tunnel ≥10 mm^2^ also was lower. In the present study, electroacupuncture improved all clinical outcomes, especially in the pain score, and these findings were relevant to the improvement of nerve conduction after electroacupuncture treatment (53). A Cochrane review also demonstrated that electroacupuncture treatment had lower pain and higher effective rate than the control group (54), which is similar to our results. Moreover, we also found that there was no significant difference between electroacupuncture treatment and the control group in terms of complications.

## 5. Current limitation

However, there are also some limitations in our study: (1) Some pooled results from included studies were strongly subjective, which may influence the results due to the different experience from doctors. (2) We only include studies reported in English and Chinese, which may lead to language bias, and this also might cause the source of heterogeneity. (3) We only consider electroacupuncture treatment as an intervention, without consideration of other details of the treatment, such as selection of acupoints and manual techniques. (4) Some results have significant heterogeneity. Although we perform the sensitivity analysis to explore the potential source of heterogeneity, there are still clinical outcomes that have not found the source of heterogeneity. Moreover, though a total of 26 randomized controlled trials with 1,698 patients were included in our study, we still needed a lot of large-sample RCTs to decrease bias and to verify the clinical outcomes. The results of this study will provide reliable and practical suggestions for acupuncture practitioners in clinical decision-making.

## 6. Conclusion

Current evidence indicated that electroacupuncture was a safe and effective therapy for CTS. In addition, electroacupuncture not only provided lower VAS pain score but also had lower scores of symptoms and function. Electroacupuncture also had better electrophysiological parameters and higher total effective rate. Based on these results, we infer that electroacupuncture was an acceptable strategy for mild-to-moderate CTS patients. However, these findings needed to be verified in further by multicenter, double-blind, and large-sample RCTs.

## Data Availability

The original contributions presented in the study are included in the article/Supplementary Material, further inquiries can be directed to the corresponding author.
